# Sorting Signals That Mediate Traffic of Chitin Synthase III between the TGN/Endosomes and to the Plasma Membrane in Yeast

**DOI:** 10.1371/journal.pone.0046386

**Published:** 2012-10-03

**Authors:** Trevor L. Starr, Silvere Pagant, Chao-Wen Wang, Randy Schekman

**Affiliations:** 1 Howard Hughes Medical Institute, Department of Molecular and Cell Biology, University of California, Berkeley, California, United States of America; 2 Graduate Group in Microbiology, University of California, Berkeley, California, United States of America; Institute of Molecular and Cell Biology, Singapore

## Abstract

Traffic of the integral yeast membrane protein chitin synthase III (Chs3p) from the *trans*-Golgi network (TGN) to the cell surface and to and from the early endosomes (EE) requires active protein sorting decoded by a number of protein coats. Here we define overlapping signals on Chs3p responsible for sorting in both exocytic and intracellular pathways by the coats exomer and AP-1, respectively. Residues 19DEESLL24, near the N-terminal cytoplasmically-exposed domain, comprise both an exocytic di-acidic signal and an intracellular di-leucine signal. Additionally we show that the AP-3 complex is required for the intracellular retention of Chs3p. Finally, residues R374 and W391, comprise another signal responsible for an exomer-independent alternative pathway that conveys Chs3p to the cell surface. These results establish a role for active protein sorting at the *trans*-Golgi *en route* to the plasma membrane (PM) and suggest a possible mechanism to regulate protein trafficking.

## Introduction

The selective packaging of transmembrane cargo proteins into transport vesicles requires specific recognition of signals within the cytosolic part of the cargo by coat protein complexes. Such a mechanism has been described for almost all intracellular vesicular transport events. Selective export of membrane cargo proteins from the endoplasmic reticulum (ER) is mediated by interaction of the Sec24 subunit of the COPII coat with diverse di-acidic or hydrophobic motifs, and retrograde transport from the *cis*-Golgi is driven by the recognition of C-terminal di-lysine motifs by the COPI coat complex [1], [2]. Later in the secretory pathway, the adaptor protein complexes (APs), Golgi-localized gamma ear-containing ARF-binding proteins (GGAs), and epsin-related proteins sort transmembrane proteins between the TGN, endosomes, and lysosomes [3]–[5]. These transport events are usually mediated by tyrosine-based sorting signals or di-leucine-based signals (for review [6]).

In addition to the conserved GGA adaptors, which transport proteins from the TGN to late endodomes, there are four AP complexes in mammalian cells and three in *Sacharomyces cerevisiae* (*S. cerevisiae*). All are composed of four subunits: one small subunit (σ1–4), one medium subunit (μ1–4), and two large subunits (β1–4 and γ1, α2, δ3, or ε4) [7]. The AP-1 complex mediates trafficking between the TGN and endosomes [3], AP-2 mediates endocytosis [8], AP-3 directs proteins toward lysosomes in mammalian cells and the vacuole in yeast [9], [10], and AP-4 may be involved in lysosomal and/or basolateral protein sorting [11], [12] and in the selective transport of cargo from the TGN to the endosome [13]. The types of signals to which AP complexes and GGAs bind is best characterized in mammalian cells. The μ subunits of the APs bind to YXXΦ sorting signals (where Φ represents bulky hydrophobic amino acids) and a combination of the σ and γ (AP-1), α (AP-2), or δ (AP-3) subunits bind to [D/E]XXXL[L/I/V] signals [14]–[16]. The GGA proteins bind to a smaller di-leucine signal, DXXLL, in which there are two residues between the critical acidic and di-leucine residues instead of three [6]. Yeast adaptors recognize sorting determinants that are conserved and non-conserved with respect to their mammalian counterparts. In yeast and mammalian cells, AP-3 acts on [D/E]XXXL[L/I/V] signals [17], [18]. Conversely, the yeast GGA proteins lack the binding pocket required to interact with DXXLL signals and instead use ubiquitin as a signal [19], [20]. Finally, at least two AP-1-dependent signals have been described thus far in yeast. The first is found in the protein DPAPA, encoded by *STE13*, and is of the non-canonical sequence, MSASTHSHKRKN [21]. The second is found in the vacuolar membrane protein Sna2p and is of the sequence YSHL [22].

Protein transport from the TGN to the PM is mediated by a less well-understood process, as there are limited examples of specific recognition of cytosolic signals within cargos by coat protein complexes. In some cases export signals may not exist and traffic through this route may be by default or governed by the length of the transmembrane (TM) span, and in turn dictated by differences in the lipid composition of the TGN and PM [23]. Increasing or decreasing the length of the TMs of single transmembrane domain proteins, or the use of synthetic sequences of different lengths fused to soluble reporters showed that long TM domains direct PM localization in yeast and mammalian cells [24], [25]. In other cases, however, signals are necessary for efficient transport from the TGN to the PM.

In mammalian cells, transport of Kir2.1, a member of the superfamily of inward rectifier potassium channels, constitutes a key example of selective export at the TGN for the surface expression of a native protein. In this case, two independent signals in the primary structure of the protein each contribute to cell-surface transport: basic residues in the cytosolic N terminus and a tyrosine motif within the cytosolic C terminus are both necessary for delivery to the PM. The coat proteins that recognize these motifs have not been identified [26]. Recently a non-linear signal in the quaternary structure of Kir2.1 has been shown to be required for PM localization and interaction with the AP-1 complex, thus adding a greater level of complexity to signal-regulated delivery of proteins from the TGN to the PM [27].

The exomer of *S. cerevisiae* is a novel coat protein complex involved in trafficking proteins from the TGN to the PM. This complex is composed of the proteins Chs5p and four paralogous ChAps (*C*hs5p-*A*rf1p binding *p*roteins), and is required for trafficking the type I cell-cell fusion protein Fus1p to the PM during the mating response [28]. Characterization of exomer-mediated trafficking of Fus1p revealed the presence of a novel linear signal in the cytosolic portion of the protein which is of the sequence IXTPK and is required for interaction of Fus1p with exomer and its trafficking to the PM during the mating response [28].

The exomer complex has also been shown to be required for transport of the major chitin synthase Chs3p from the TGN to the PM in a cell cycle regulated manner [29]–[31]. At steady state, Chs3p is localized at the bud neck but is also maintained in an intracellular reservoir by continuously cycling between the TGN and the EE [32], [33]. Cells harboring individual deletions of *CHS5* or the ChAP *CHS6* or double mutations in the ChAPs *BCH1* and *BUD7* fail to traffic Chs3p to the cell surface [34], [35]. Additionally, Chs3p has been shown to physically interact with the exomer complex using both in vivo crosslinking experiments and in vitro pull down assays [30], [31].

The intracellular pool of Chs3p is maintained by its cycling between the TGN and endosomes, dependent on the action of the clathrin adaptors AP-1, Gga1p/Gga2p, and the epsin related proteins Ent3p/Ent5p [33], [36]. In cells carrying mutations in these adaptors, Chs3p reaches the plasma membrane from the TGN or the EE by at least one alternative exocytic pathway that bypasses the requirement for exomer [33]. The sequences within Chs3p required for intracellular trafficking, exomer-dependent trafficking, and alternative exocytic transport remain to be identified. Here, we demonstrate that Chs3p contains specific information that is necessary for its transport through the exomer pathway, intracellular AP-1-dependent pathway, and the alternative exocytic pathway. Additionally, we show that yet another AP complex, AP-3, is involved in the intracellular retention of Chs3p.

## Materials and Methods

### Growth Conditions

Yeast cultures were grown in YPD (1% yeast extract, 2% peptone, 2% glucose), or synthetic complete (SC) dropout media (0.67% nitrogen base, 2% glucose, complete drop-out supplements (Q-biogene, Carlsbad, CA)). Resistance to calcofluor (CF) was assessed by growth on SC–Ura agar plates supplemented with 50 or 100 µg/ml Fluorescent Brightener 28 (calcofluor) (Sigma Chemical Co., St. Louis, MO). To prevent precipitation of CF in the SC-Ura + CF agar plates, we adjusted the minimal medium near pH 7.0 by the addition of 0.7 M KH_2_PO_4_, pH 7.0 to a final concentration of 10%. Growth on 1/2 YPD agar medium (0.5% yeast extract, 1% peptone, 1% glucose, 1% agar) was used in the genetic selection that identified the DEESLL signal.

### Yeast Strain Construction

Strains (Table 1) were constructed either by tetrad dissection of sporulated diploid strains or by integration of disruption cassettes that were generated from plasmid templates or pre-existing chromosomal deletions [37], [38]. All allelic replacements were confirmed by PCR.

**Table 1:** Strains used in this study.

Name	Genotype	Reference
YPH499	*MATa ade2-101oc his3-Δ200 leu2-Δ1 lys2-801am trp1-Δ63 ura3-52*	[52]
JCY306	*MATa ade2 his3 leu2 lys2 trp1 ura3 chs3Δ::LEU2*	[50]
SPY06	*MATa ade2 his3 leu2 lys2 trp1 ura3 chs3Δ::LEU2 chs6Δ::KANMX*	This study
SPY21	*MATa ade2 his3 leu2 lys2 trp1 ura3 chs3Δ::LEU2 apl4Δ::TRP1*	This study
SPY10	*MATa ade2 his3 leu2 trp1 ura3 chs3Δ::LEU2 chs6Δ::HIS3 apl4Δ::TRP1*	This study
TSY49	*MATa ade2-101oc his3-Δ200 leu2-Δ1 lys2-801am trp1-Δ63 ura3-52 chs6Δ::HIS3*	This study
TSY131	*MATa prb1-1122 pep4-3 prc1-407 gal2 leu2 trp1 ura3-52 APS1::S-Tag-Tev-ZZ::KANMX*	
TSY178	*MAT? ade2-101oc his3-Δ200 leu2-Δ1 lys2-801am trp1-Δ63 ura3-52 chs6Δ::HIS3*	This study
	*apl2Δ::TRP1 apl6Δ::KANMX*	
TSY194	*MATa ade2-101oc his3-Δ200 leu2-Δ1 lys2-801am trp1-Δ63 ura3-52 chs3Δ::LEU2*	This study
	*chs6Δ::TRP1*	
TSY269	*MATa ade2-101oc his3-Δ200 leu2-Δ1 lys2-801am trp1-Δ63 ura3-52 chs6Δ::HIS3 apl6Δ::HIS5*	This study
TSY300	*MATa ade2-101oc his3-Δ200 leu2-Δ1 lys2-801am trp1-Δ63 ura3-52 chs6Δ::HIS3 apl1Δ::URA3*	This study
RSY3393	*MAT? ade2-101oc his3-Δ200 leu2-Δ1 lys2-801am trp1-Δ63 ura3-52 chs6Δ::HIS3 apl2Δ::TRP1*	[36]

### Plasmid Construction

Point mutations and deletions within *CHS3* were introduced by QuikChange Mutagenesis (Stratagene, La Jolla, CA) using primers containing the desired changes and plasmid pJC345 as a template. Plasmid pJC345 contains a copy of *CHS3* under the control of its own promoter inserted into the EcoR1/SalI sites of pRS316 [All plasmids used in this study are listed in Table 2].

**Table 2:** Plasmids used in this study.

Name	Description	Source
pJC345	EcoRI-SalI genomic fragment containing *CHS3* subcloned in pRS316	[50]
pCHS3QC1	W391R (Chs3W391R) mutation in pJC345	This study
pCHS3QC2	W391L (Chs3W391L) mutation in pJC345	This study
pCHS3QC3	W391F (Chs3W391F) mutation in pJC345	This study
pCHS3QC4	W391S (Chs3W391S) mutation in pJC345	This study
pCHS3QC5	W391A (Chs3W391A) mutation in pJC345	This study
pCHS3QC6	W391T (Chs3R374T) mutation in pJC345	This study
pCHS3QC7	W391A (Chs3R374A) mutation in pJC345	This study
pCHS3QC103	N17AQ18A (Chs3N17AQ18A) mutation in pJC345	This study
pCHS3QC133	D19A (Chs3D19A) mutation in pJC345	This study
pCHS3QC135	E20A (Chs3E20A) mutation in pJC345	This study
pCHS3QC137	E21A (Chs3E21A) mutation in pJC345	This study
pCHS3QC131	S22A (Chs3S22A) mutation in pJC345	This study
pCHS3QC106	L23A (Chs3L23A) mutation in pJC345	This study
pCHS3QC107	L24A (Chs3L24A) mutation in pJC345	This study
pCHS3QC139	R25A (Chs3R25A) mutation in pJC345	This study
pCHS3QC115	N17AQ18A (Chs3N17AQ18AW391R) mutation in pCHS3QC1	This study
pCHS3QC134	D19A (Chs3D19AW391R) mutation in pCHS3QC1	This study
pCHS3QC136	E20A (Chs3E20AW391R) mutation in pCHS3QC1	This study
pCHS3QC138	E21A (Chs3E21AW391R) mutation in pCHS3QC1	This study
pCHS3QC132	S22A (Chs3S22AW391R) mutation in pCHS3QC1	This study
pCHS3QC118	L23A (Chs3L23AW391R) mutation in pCHS3QC1	This study
pCHS3QC119	L24A (Chs3L24AW391R) mutation in pCHS3QC1	This study
pCHS3QC144	R25A (Chs3R25AW391R) mutation in pCHS3QC1	This study
pD15	chs3p deleted of N-terminal 15 AA (Δ15CHS3)subcloned in p416MET25	This study
pD15WR	W391R (Δ15CHS3(W391R)) mutation in pD15	This study
pD25	chs3p deleted of N-terminal 25 AA (Δ25CHS3) subcloned in p416MET25	This study
pD25WR	W391R (Δ25CHS3(W391R)) mutation in pD25	This study
pJC322	N-terminal 171AA of Chs3p GST-tagged (Chs3(1–170)) in pGEX-2T	[50]
pCHS3QC164	D19AE21A (Chs3(1–170)D19AE21A) mutation in pJC322	This study
pCHS3-2GST	Second cytosolic loop of Chs3p GST-tagged (Chs3(224–451)) in pGEX-2T	This study
pTS11	L24H (Chs3-L24H) mutation in pJC345	This study
pTS13	L24F (Chs3-L24F) mutation in pJC345	This study
pTS15	L24P (Chs3-L24P) mutation in pJC345	This study
pTS18	D19N (Chs3-D19N) mutation in pJC345	This study
pTS166	DEESLLΔ (Chs3-DEESLLΔ) mutation in pJC345	This study

### Quantitative Chitin Assays

The protocol used for quantitative chitin assays was largely adopted from Bulik et. al. [39]. Yeast cultures were grown in YPD to saturation, after which 35–50 mg of cells were collected for analysis. Yeast cells were lysed in 500 ul of 6% KOH at 95°C for 90 min. Cell wall material was sedimented for 10 min at top speed in a microcentrifuge. Pellets were rinsed with 1 ml 1× PBS, then 500 ul McIlvaine's buffer (63% 0.2 M Na_2_HPO_4_, 37% 0.1 M citric acid), pH 6.0, and then resuspended in 100 ul McIlvaine's buffer, pH 6.0, by sonication with a microtip sonicator. Chitin in the cell wall material was digested by addition of 8 ul 7 mg/ml chitinase from *Trichoderma viride* (*T. viride*) (Sigma Chemical Co., St. Louis, MO #C8241) at 37°C for 18 h. After incubation, the remaining cell wall was sedimented at top speed for 10 min. Quantification of the liberated GlcNAc was achieved with a colorimetric assay. Aliquots (10 ul) of sample were mixed with 10 ul of 0.27 M sodium borate, pH 9.0, and heated at 99°C for 10 min and then cooled on ice for 5 min. Immediately before use, DMAB solution (1 g p-dimethylaminobenzaldehyde in 1.25 ml concentrated HCL and 8.75 ml glacial acetic acid) was diluted 1∶10 into glacial acetic acid and then 100 ul added to each tube. Tubes were incubated at 37°C for 20 min to allow development of color. Tubes were briefly chilled on ice and 90 ul of each reaction were aliquotted to a microtiter plate. The intensity of the reaction was measured at OD570 in a Dynatech MR5000 microtiter plate reader. Experimental samples were compared against a standard curve using reactions with pure GlcNAc solutions (0 mM, 0.125 mM, 0.25 mM, 0.5 mM, 1 mM, and 2 mM), and calculated as nmol of GlcNAc/mg cells. For each experiment, two to three replicates were conducted for each strain and used to determine the average amount of GlcNAc released per strain. Averages were normalized against wt values and graphed.

### Sucrose Gradient Fractionation

The analysis of organelles by sucrose gradient fractionation was performed as described [40]. In brief, 10 OD600 units of mid-log cells were harvested by centrifugation and washed with ice-cold 20 mM N_a_N_3_/20 mM KF. Cells were digested with lyticase and the resulting spheroplasts were lysed by osmotic shock with 0.35 ml of lysis buffer (5% sucrose in 20 mM triethanolamine, pH 7.2, 1 mM EDTA, 1 mM phenylmethylsulfonyl fluoride). Unlysed cells were removed by centrifugation (500× *g* for 2 min). Total cell lysates (0.2 ml) were overlaid on a step sucrose/EDTA gradient (0.2 ml 55%, 0.5 ml 45%, 0.4 ml 30% sucrose (w/w) in 20 mM triethanolamine, pH 7.2, 5 mM EDTA) and centrifuged at 55,000 rpm in a TLS55 rotor (Beckman) for 2.5 h. Fractions (0.2 ml) were collected manually from the top, solubilized in 1% SDS at 55°C for 10 min, and analyzed by SDS-PAGE and immunoblotting with antibodies against Chs3p (Schekman lab), Pma1p (F. Portillo, Universidad Autonoma de Madrid, Spain) and Tlg1p (H. Pelham, Medical Research Council, Cambridge, UK).

### Microscopy

Yeast cultures were grown to mid-log phase in SC-Ura and then fixed by addition of 37% formaldehyde to a final concentration of 4%, followed by incubation on ice for 30 min. Fixed cells were washed twice with 1 ml dH_2_O and resuspended in 250 ul of a 50 ug/ml calcofluor solution for 30 min on ice. Cells were washed twice with 1 ml dH_2_O and visualized with a DAPI filter on a Nikon epifluorescence microscope. Images were captured with a CCD camera and processed with Adobe Photoshop.

### Chs3p/Chs5p Binding Experiments

*Escherichia coli* (*E. coli*) BL21(DE3)pLysS cells (Stratagene, La Jolla, CA) harboring plasmids containing GST-Chs3 fragments were grown in 500 ml cultures to an OD600 of 0.5–1.0, at which time IPTG was added to a final concentration of 250 uM. Soluble cell lysates (15 ml) were incubated with 2 ml glutathione agarose at 4°C for 3 h. Beads were washed three times with 10 ml PBSG buffer (phosphate buffer, pH 7.3, 140 mM NaCl, 2.7 mM KCl, 10% glycerol) followed by one additional wash in 10 ml HKSG (50 mM HEPES, pH 7.4, 50 mM KOAc, 200 mM sorbitol, 10% glycerol). Bound proteins were eluted with 12 ml HKSG buffer containing 10 mM reduced glutathione. A total of 10 fractions were collected and protein concentration was measured by the Bradford assay. For all proteins purified for this experiment, more than 2 mg of GST-tagged protein were recovered with at least 90% purity. To test if these Chs3p fragments interacted with Chs5p or the exomer complex, we expressed and purified His-Chs5 and His-exomer complex from baculovirus infected culture cells (Wang *et al*., 2006). Aliquots of 100 ul His-Chs5p and His-exomer complex were immobilized on Ni-NTA beads and incubated with 150 ug/ml purified GST-Chs3 fragments at RT for 30 min. The beads were first washed with 1.5 ml B88G+Tween (20 mM HEPES, pH 7.4, 150 mM KOAc, 250 mM sorbitol, 5 mM MgCl2, 10% glycerol, 1% Tween 20), and then 4 ml B88G in a 15 ml tube, followed by one additional wash with 1.5 ml B88G. Beads were resuspended in 400 ul MURB (50 mM Na_2_HPO_4_, 25 mM MES, pH 7.0, 1% SDS, 3 M urea, 0.5% β-mercaptoethanol) and bound proteins were eluted by heating in boiling water for 5 min, followed by SDS-PAGE and coomassie blue staining.

### Tandem Affinity Purification (TAP) Tag co-purification of AP-1 and Chs3p

Two liters of each strain were grown overnight in SC-Ura medium to an OD 600 of ∼1.0. Cells (1500 OD_600_) were centrifuged, washed with 50 ml dH2O, and resuspended in 40 ml of spheroplasting pre-treatment buffer (100 mM Tris, pH9.4, 40 mM βme) and incubated at 30°C for 10 min. Treated cells were resuspended in 40 ml of spheroplasting buffer (0.7 M sorbitol, 0.75× YPD, 20 mM Hepes, pH 7.5, and 4 mM βme) with lyticase (2 ul/OD cells) and cells were converted to spheroplasts during a 45 min incubation at 30°C for. Spheroplasts were washed in 40 ml cross-linking buffer (0.7 M sorbitol, 20 mM Hepes pH 7.5, and 125 mM KOAc) and then reuspended in 30 ml fresh cross-linking buffer. A 100 mM stock of DSP was prepared in DMSO immediately before use. DSP was added to the spheroplast suspension at a final concentration of 5 mM and incubated at room temperature for 30 min. To quench any remaining DSP, 2 M Tris, pH7.5, was added to a final concentration of 100 mM and the mixture was incubated for 15 min at room temperature. Treated spheroplasts were resuspended in 15 ml of lysis buffer (50 mM Hepes, pH 7.5, 1% Triton X-100, 0.4 M NaCl and protease inhibitors) and lysis was achieved with 15 strokes of a dounce homogenizer. Lysates were incubated on ice for 30 min to allow extraction of membrane proteins. Unbroken cells and large debris were removed from the lysates by centrifugation at 500× g for 10 min. The supernatant was further clarified by centrifugation at 13,000× g for 20 min. Proteins that bind nonspecifically to agarose beads were removed by incubation of the lysates with 2 ml CL-6B agarose at 4°C for 30 min. The resulting lysates were incubated with a 100 ul bed volume of IgG Sepharose for 3 h at 4°C. Beads were washed with 10 ml of lysis buffer three times. AP-1-TAP was eluted from the beads by incubation with TEV protease overnight at 4°C. To achieve the second round of purification, we incubated the elution sample with a 30 ul bed volume of S-protein agarose for 3 h at 4°C. Beads were washed with 1 ml of lysis buffer 5 times. Proteins were eluted with 30 ul of 2× sample buffer containing 100 mM DTT at 55°C for 10 minutes. Proteins were resolved on 10% polyacrylamide gels and total protein was visualized by staining with sypro-red, prior to transfer to PVDF membranes. Immunoblot analysis to determine the amount of co-purified Chs3 was carried out using anti-Chs3 antibodies.

## Results

### Identification of a Sorting Signal Required for the Intracellular Retention of Chs3p

We have previously reported that a number of clathrin adaptor proteins are involved in transporting Chs3p between the TGN and endosomes [33], [36]. To better understand how these intracellular transport pathways are achieved we conducted a genetic selection to identify the sorting signals in Chs3p that mediate these events. We relied on the fact that mutations that inhibit Chs3p intracellular trafficking cause Chs3p to engage in an exomer-independent alternative exocytic pathway [33]. Deletion of the exomer component *CHS6* blocks Chs3p localization to the PM, which in turn results in greatly reduced levels of chitin in the cell wall. Phenotypically, *chs6Δ* strains are resistant to the toxic effects of the chitin binding drug calcofluor and are sensitive to growth on ½ YPD, a hypo-osmotic medium. Restoration of Chs3p to the PM of a *chs6Δ* strain, via the alternative exocytic pathway, results in cells that are sensitive to calcofluor and resistant to growth on ½ YPD [33]. We reasoned that mutations in the sorting signal required for intracellular retention of Chs3p would allow Chs3p to localize to the PM of *chs6Δ* cells, conferring calcofluor-sensitive and ½ YPD-resistant phenotypes.

**Figure 1:**
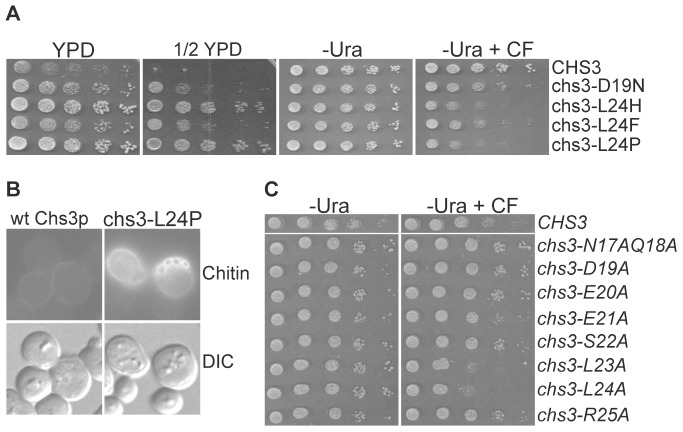
Chs3p residues 19DEESLL24 constitute an intracellular retention signal. (A) Ten-fold serial dilutions of *chs3Δ chs6Δ* cells (TSY194) expressing point mutant alleles of *CHS3* that define 19DEESLL24 as an intracellular retention signal. Dilutions were spotted on YPD, ½ YPD, SC-Ura (-Ura) and SC-Ura +50 ug/ml calcofluor (-Ura +CF). (B) Chitin staining of TSY194 expressing the *chs3-L24P* mutant allele. (C) Ten-fold serial dilutions of TSY194 expressing alanine-scanning point mutants of Chs3p residues 17–25.

To carry out the selection, we randomly mutagenized a low copy plasmid that carried wt *CHS3*, under the control of its own promoter, by propagating the plasmid in XL1-Red *E. coli* cells (Stratagene, La Jolla, CA), a strain of *E. coli* that is deficient in DNA repair pathways. Mutagenized plasmid pools were transformed into a *chs3Δ chs6Δ* strain, and transformants were replica-plated onto ½ YPD. Colonies resistant to this medium were further tested for sensitivity to medium containing calcofluor. Using this method, four alleles of *CHS3* that conferred resistance to ½ YPD and sensitivity to calcofluor in the *chs3Δ chs6Δ* strain were isolated (Figure 1A). The mutations coded for the following amino acid substitutions: D19N, L24H, L24F, and L24P. These residues define the sequence 19DEESLL24, in the N-terminus of Chs3p, which matches the consensus sequence for AP-dependent sorting signals, [D/E]XXXL[L/I/V] [6].

To confirm that the mutant variants of Chs3p were able to localize to the PM of *chs3Δ chs6Δ* cells, we assayed chitin in the cell wall by staining cells with calcofluor (Figure 1B). During the cell cycle Chs3p synthesizes a ring of chitin around the mother-bud neck, marking sites of current and previous cell division. In cells expressing Chs3p at the PM, staining with calcofluor reveals numerous bright chitin rings, whereas in cells that lack PM-localized Chs3p, such as *chs6Δ* cells expressing wt Chs3p, chitin rings are absent. Figure 1B shows that, as expected, chitin was absent from the cell walls of *chs3Δ chs6Δ* cells harboring a plasmid expressing wt *CHS3*, but present in cells expressing the Chs3-L24P point mutant, confirming that the DEESLL signal is required for the intracellular retention of Chs3p.

In signals of this type the most critical residues are the first acidic residue and the di-leucine residues [6]. We tested if this was the case for the DEESLL signal by conducting alanine-scanning mutagenesis of nucleotides that correspond to amino acid residues 17 through 25 of Chs3p, and assayed for the ability of these mutants to confer a calcofluor sensitive phenotype to *chs3Δ chs6Δ* cells (Figure 1C). Of all the alanine substitutions tested, only L23A and L24A resulted in calcofluor sensitivity when introduced in *chs3Δ chs6Δ* cells (Figure 1B). Interestingly, the D19A mutation had no obvious effect, even though the substitution D19N was isolated in our ½ YPD selection (Figure 1A). These results indicate that the aspartic acid residue plays only a minor role in the intracellular trafficking of Chs3p, whereas the leucine residues are essential.

### The DEESLL Signal is Required for a Physical Interaction between AP-1 and Chs3p

**Figure 2:**
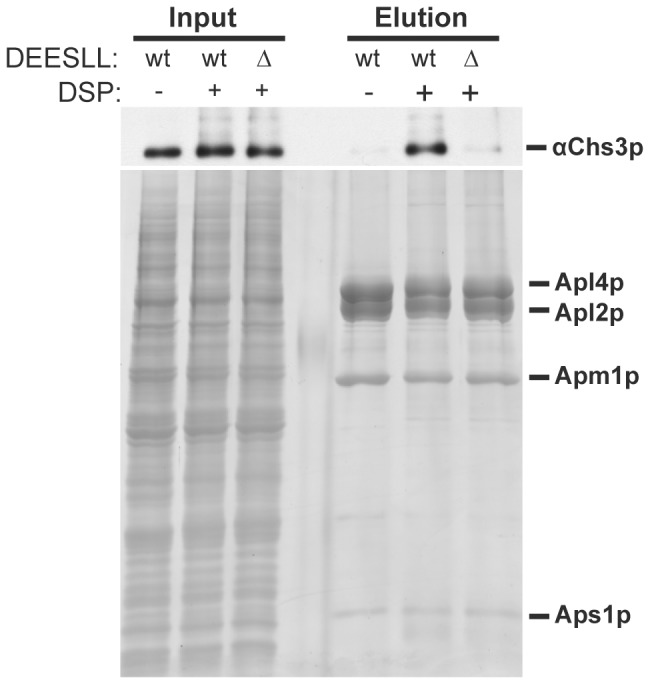
The DEESLL signal is required for a physical interaction between AP-1 and Chs3p. Cells of the genotype *chs3Δ pep4Δ prb1Δ prc1Δ APS1-TAP* (TSY131), expressing either wt Chs3p or Chs3-DEESLLΔ were grown overnight, converted to spheroplasts, and treated with the crosslinking agent DSP prior to lysis and purification of the AP-1 complex via a TAP tag. Samples were separated on a 10% polyacrylamide gel, which was visualized with sypro red stain prior to transfer to a PVDF membrane for evaluation by imunoblot. Shown is a western for Chs3p and a total protein stain for the AP-1 purification. Note that Chs3-DEESLLΔ does not copurify with AP-1.

The DEESLL signal matches the consensus motif for AP binding, and we have previously shown that AP-1 and Chs3p physically interact [6], [36]. Thus, it seemed likely that AP-1 might interact with this sequence to facilitate intracellular transport of Chs3p. To test this hypothesis, we utilized a strain in which CHS3 was deleted and the smallest subunit of AP-1 (Aps1p) was fused to a tandem affinity purification tag [36]. Into this strain we transformed a plasmid expressing either wt Chs3p or Chs3-DEESLLΔ. We then added the crosslinking agent DSP to living cells to stabilize the interaction between AP-1 and the Chs3p variants and subsequently purified AP-1 from yeast cells. As demonstrated previously [36], wt Chs3p co-purified with AP-1 (Figure 2). In contrast to the result with wt Chs3p, Chs3-DEESLLΔ failed to co-purify with AP-1, demonstrating that the DEESLL signal is required for the physical interaction of Chs3p and AP-1 (Figure 2).

### The AP-3 Complex is Required for the Intracellular Retention of Chs3p

**Figure 3:**
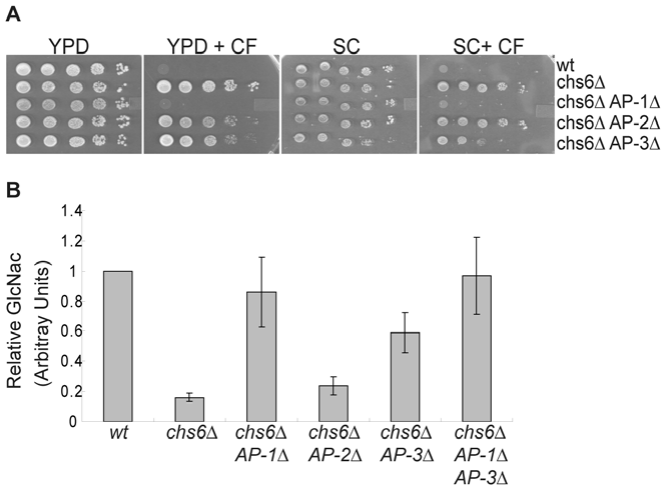
AP-3 is required for the intracellular retention of Chs3p. (A) Ten-fold serial dilutions of wt (YPH499), *chs6Δ* (TSY49), *chs6Δ apl2Δ* (RSY3393), *chs6Δ apl1Δ* (TSY300) and *chs6Δ apl6Δ* (TSY269) cells on YPD, YPD + 50 ug/ml calcofluor (YPD + CF), synthetic complete medium (SC), and synthetic complete medium +50 ug/ml calcofluor (SC + CF). (B) An average of 5 quantitative chitin assays. Cultures were grown to saturation and cell walls were isolated by lysing 35–50 mg of cells in 6% KOH. Chitin was digested with chitinase from *T. viride* and the resulting GlcNAc was quantified. For each assay the results of 2–3 replicates of each strain were averaged and normalized against the wt level of chitin. Five separate normalized experiments were then averaged together.

We previously reported that AP-2 and AP-3 do not have roles in transporting Chs3p [33]. However, during the course of our studies we re-examined this issue. We introduced mutations in the β2 subunit of AP-2 (*apl1Δ*) and the β3 subunit of AP-3 (*apl6Δ*) into a *chs6Δ* strain and tested the ability of these mutations to confer calcofluor sensitivity (Figure 3A). When grown on rich YPD medium containing 50 ug/ml calcofluor, strains harboring the (*AP-2Δ*) and (*AP-3Δ*) mutations exhibited unaltered calcofluor-resistance. However, on synthetic complete (SC) minimal medium the *chs6Δ AP-3Δ* cells were partially calcofluor-sensitive.

To resolve the discrepancy between the YPD and SC media, we conducted quantitative chitin assays to determine the extent to which chitin was restored to the cell walls of these strains. To conduct these assays, we lysed cells in an alkaline solution from which cell wall material was recovered in an insoluble fraction. This material was digested with chitinases to yield N-acetyl-glucosamine (GlcNAc), which was quantified using a colorimetric assay. As expected, a *chs6Δ* mutant, where Chs3p is not targeted to the PM, had low levels of chitin in the cell wall, compared to wt (Figure 3B). The *chs6Δ AP-2Δ* strain had low levels of chitin equivalent to the parental *chs6Δ* strain, confirming that AP-2 does not play a significant role in trafficking Chs3p. Conversely, the *chs6Δ AP-1Δ* strain had a level of chitin that was much greater than that of the *chs6Δ* single mutant, and interestingly, the *chs6Δ AP-3Δ* strain contained a level of chitin that was intermediate between that of the *chs6Δ* single mutant and the *chs6Δ AP-1Δ* mutant. This result indicated that AP-3 is important for the intracellular transport of Chs3p, but plays a less significant role than AP-1. The lower level of chitin in *chs6Δ AP-3Δ* cells may explain why this strain did not show obvious calcofluor sensitivity on YPD. Our general experience with chitin assays is that growth in SC medium leads to higher levels of chitin in the cell wall compared to growth in YPD. The reason for this difference is unknown, but may explain why the *chs6Δ AP-3Δ* strain showed partial calcofluor sensitivity on SC medium but not YPD.

We also wished to determine if AP-1 and AP-3 function in the same or distinct pathways of Chs3p transport. We reasoned that if these adaptors function in the same pathway, the amount of chitin restored to the cell wall of *chs6Δ AP-1Δ AP-3Δ* cells would not exceed that of the *chs6Δ AP-1Δ* mutant. Conversely, if these adaptors function in distinct pathways of Chs3p transport, the effect of the APΔ mutations should be additive, resulting in more chitin in the cell walls of the *chs6Δ AP-1Δ AP-3Δ* mutant than the *chs6Δ AP-1Δ* or the *chs6Δ AP-3Δ* mutant. Figure 3 shows that, when averaged from five different experiments, we did not observed a significant enhancement of chitin levels in the triple mutant compared to the double mutant. Based on these findings we conclude that AP-1 and AP-3 may serve overlapping functions in transport of Chs3p.

Finally, it is important to note that at a higher calcofluor concentration (100 ug/ml) the *chs6Δ AP-2Δ* strain was sensitive to growth on YPD + calcofluor. The quantitative chitin assay in Figure 3 demonstrated that this strain had a low level of chitin, therefore, Chs3p does not traffic to the PM of this strain. We presume that the sensitivity of this strain to high calcofluor concentrations may be caused by other defects imparted by the *apl1Δ* mutation. Taken together we conclude that AP-2 plays no significant role in trafficking Chs3p, whereas AP-3 plays an important, although less significant role than AP-1 in Chs3p transport.

### Mutations in Residues R374 and W391 Impair the Transport of Chs3p to the PM through the Alternative Exocytic Pathway

**Figure 4:**
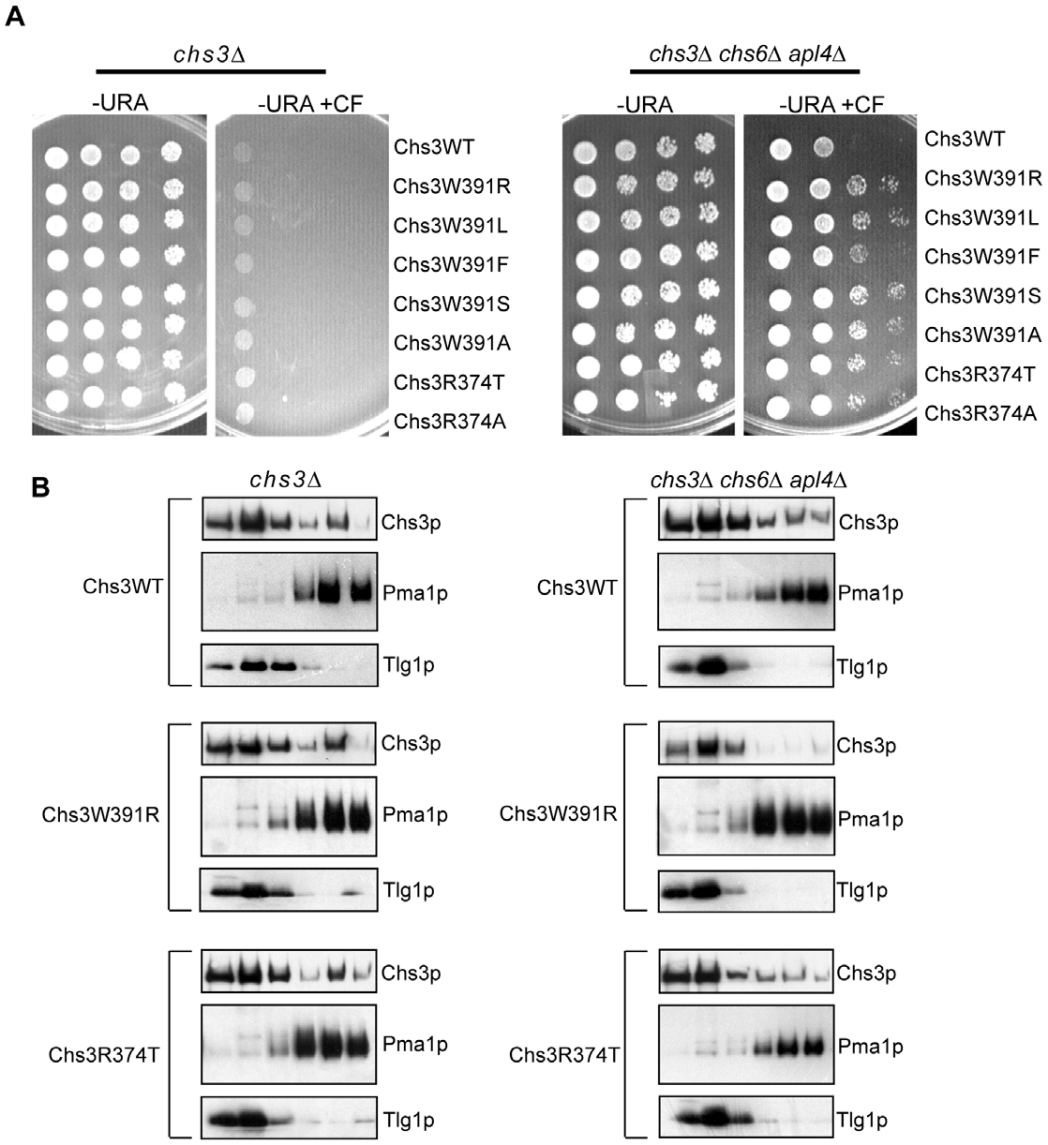
Mutations in residues R374 and W391 specifically impair access to the alternative exocytic pathway. (A) Ten-fold serial dilutions of *chs3Δ* (RSY1699) or *chs3Δ chs6Δ AP-1Δ* (SPY10) cells expressing alanine-substitution mutants of *CHS3* were spotted onto synthetic complete medium -Ura + 100 ug/ml calcofluor (-Ura + CF). (B) Subcellular fractionation of wt Chs3, Chs3-W391R and Chs3-R374A expressed in *chs3Δ* and *chs3Δ chs6Δ AP-1Δ* cells on step sucrose/EDTA gradients. Total membranes from spheroplasts were separated on a step sucrose/EDTA gradient. The protein composition of fractions obtained from differential centrifugations and sucrose gradients were analyzed by SDS/PAGE and immunoblotting (PM marker: Pma1p; Golgi/EE markers: Tlg1p).

In this and previous studies we relied on the transit of Chs3p through the alternative exocytic pathway to characterize the role of coat protein adaptors and the DEESLL signal in the intracellular trafficking of Chs3p [33], [36]. To better understand the nature of the alternative exocytic pathway, we conducted a genetic screen to isolate *CHS3* alleles specifically unable to access this route. As before, this selection was based on the calcofluor phenotype. As noted previously, *chs6Δ AP-1Δ* cells are sensitive to calcofluor. We reasoned that a mutation in the signal required for transit of Chs3p through the alternative exocytic pathway would confer calcofluor-resistance to *chs6Δ AP-1Δ* cells. A library of randomly mutagenized *chs3* was created by error-prone PCR and introduced into *chs3Δchs6Δ AP-1Δ* (*apl4*Δ) cells on a low copy plasmid, under the control of its own promoter. Clones from this library, producing Chs3p unable to reach the PM through the alternative exocytic pathway, were selected for their calcofluor-resistant phenotype. Plasmids from calcofluor-resistant transformants were recovered and introduced into a *chs3Δ* strain. We selected for further characterization only those clones that were able to complement the calcofluor-resistant phenotype of *chs3Δ*. The ability to rescue the *chs3Δ* phenotype indicated that the mutated copy of *CHS3* encoded a stable and active Chs3p protein that was able to exit the ER and, more importantly, traffic to the PM through the normal exomer-dependent pathway. Each plasmid recovered in the initial screen contained multiple mutations in the *CHS3* sequence, but after each mutation was individually reproduced by site-directed mutagenesis, a single mutation, W391R, conferred the expected phenotype: calcofluor resistance in *chs3Δ chs6Δ AP-1Δ* cells and calcofluor sensitivity in *chs3Δ* cells (Figure 4A).

The W391R mutation represents a dramatic substitution, which could induce a conformational change that would impair the accessibility of a signal situated nearby. To discriminate between these two possibilities, we tested the effect of less drastic substitutions at W391. Whereas the most conservative substitution, W391F, conferred an intermediate phenotype, W391A, W391L, and W391S, when expressed in the *chs3Δ chs6Δ AP-1Δ* strain, presented a calcofluor-resistant phenotype comparable to that of W391R (Figure 4A).

The amino acid residues D(387)LLD(390), found immediately upstream of W391, resemble a canonical di-leucine signal. The potential involvement of L389 could not be assessed because L389A and L389S mutations destabilized the Chs3p protein. However, mutation of any of the other residues in this sequence (D387A, D387V, D387S, D387T, L388A, D390A, D390L and D390F) did not confer calcofluor-resistance to a *chs3Δ chs6Δ AP-1Δ* strain, which demonstrated that these residues were not part of a signal promoting access of Chs3p to the alternative exocytic pathway. These data support the hypothesis that W391 acts as a direct sorting determinant rather than indirectly disrupting a nearby signal when altered to W391R.

The Eps15 homology (EH) domains, found in many proteins involved in different transport events between endosomes and the PM [41]–[43], contain a binding pocket for Asn-Pro-Phe (NPF) motifs, where a conserved tryptophan residue within the pocket is absolutely necessary for NPF binding [44]. In addition to this critical Trp, another conserved residue (R/K) located 16 residues upstream from the Trp also plays a critical role in NPF binding. Although Chs3p does not contain a canonical EH domain, an Arg residue is located 17 residues upstream of the critical W391. We, therefore, examined the role of R374 in promoting access of Chs3p to the alternative exocytic pathway. As with the substitutions for W391, both R374A and R374T showed calcofluor resistance when introduced in the *chs3Δ chs6Δ AP-1Δ* strain and a calcofluor-sensitive phenotype in the *chs3Δ* strain (Figure 4A), suggesting that R374 and W391 were part of the same signal and that this signal was specific to the alternative exocytic pathway.

To test the Chs3p traffic defect of these mutants, we performed membrane fractionation on sucrose/TEA gradients under conditions where intracellular endosomal/Golgi membranes were separated from the PM (Figure 4B). For these experiments we compared the localization of either wt Chs3p, Chs3W391R or Chs3R374A in *chs3Δ* or *chs3Δ chs6Δ AP-1Δ* cells. In *chs3Δ* cells ∼40% of wt or the mutant variants of Chs3p co-fractionated with the PM marker Pma1p, which confirmed the observation that the mutants were able to access the normal exomer-dependent pathway. Conversely, in the *chs3Δ chs6Δ AP-1Δ* strain, ∼30% of wild type Chs3p was present in the PM fractions, whereas Chs3W391R was not detected in those fractions and only ∼15% of the Chs3R374A mutant protein co-fractionated with Pma1p. These localization results supported the phenotypic observation that Chs3W391R and Chs3R374A mutant proteins were deficient in traversing the alternative exocytic route.

### Identification of a Signal Required for the Exomer-Dependent Transport of Chs3p

Having identified a signal that mediates transport of Chs3p via the alternative exocytic pathway, we applied a similar approach in an attempt to isolate *CHS3* alleles impaired for transport to the PM through the exomer pathway. However, we were unable to isolate point mutant alleles by this approach, and therefore chose truncation analysis as an alternative. Deletion of a region between amino acids 15 and 25 identified a segment essential for normal sorting of Chs3p.

**Figure 5:**
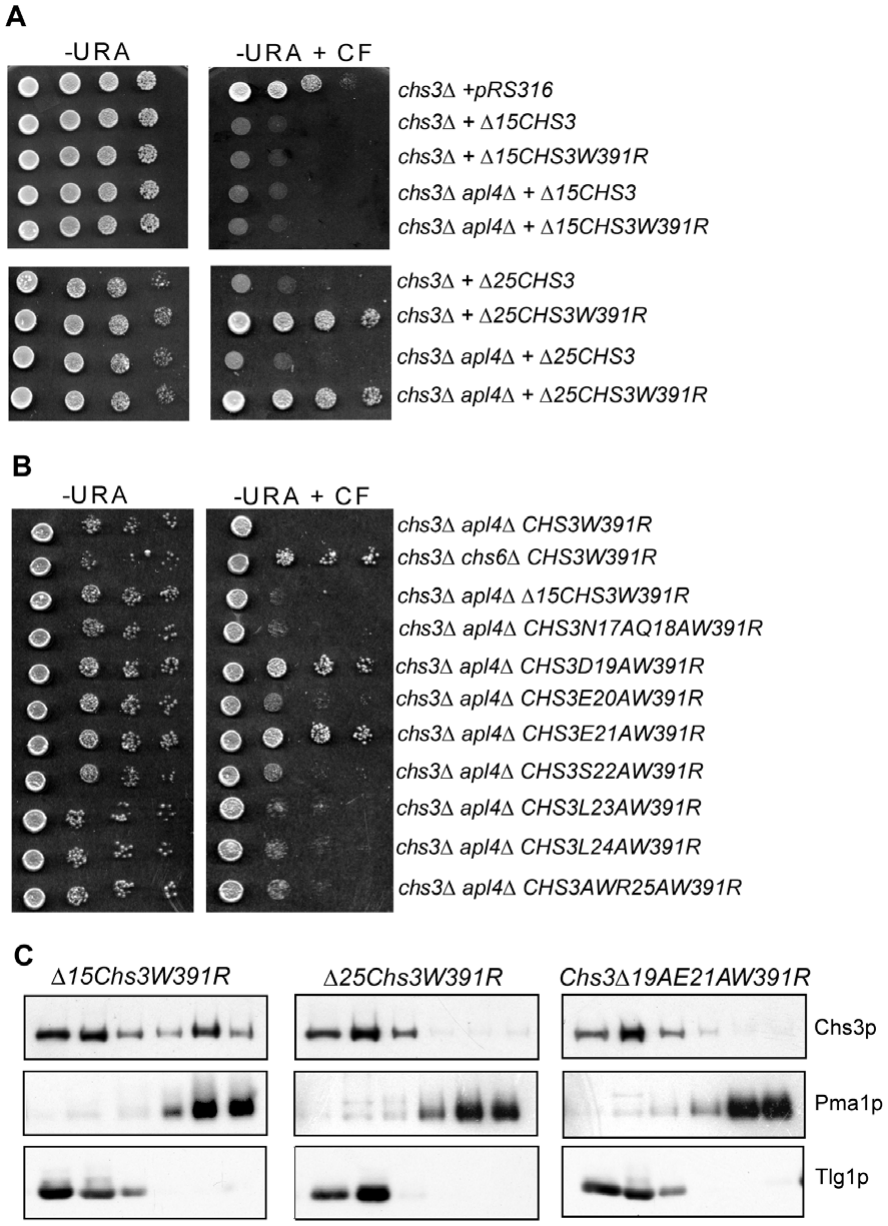
Mutations of D19 and E21 result in intracellular retention of Chs3p. (A) Ten-fold serial dilutions of *chs3Δ* (RSY1699) or *chs3Δ AP-1Δ* (SPY21) cells expressing N-terminal deletion mutants of *CHS3*, with and without the W391R substitution, were spotted onto synthetic complete medium -Ura plates +100 ug/ml calcofluor (-Ura + CF). (B) Ten-fold serial dilutions of *chs3Δ AP-1Δ* cells expressing alanine substitution mutants from residue 16 to residue 25 of Chs3-W391R were spotted onto - Ura + CF. (C) Subcellular fractionation of Δ15Chs3-W391R, Δ25Chs3-W391R and Chs3-D19AE21AW391R expressed in *chs3Δ AP-1Δ* cells on step sucrose/EDTA gradients. Total membranes from spheroplasts were separated on a step sucrose/EDTA gradient. The protein composition of fractions obtained from the sucrose gradients was analyzed by SDS/PAGE and immunoblotting (PM marker: Pma1p; Golgi/EE markers: Tlg1p).

We examined which exocytic pathway, the exomer pathway or the alternative exocytic pathway, N-terminally truncated forms of Chs3p use for their transport to the PM. Deletion of the N-terminal 15 or 25 amino acids produced stable and active proteins, capable of leaving the ER and of being transported to the PM, as shown by the calcofluor-sensitive phenotype they conferred on *chs3Δ* cells (Figure 5A). It was unclear, however, if these proteins arrived at the PM strictly via the exomer pathway, the alternative exocytic pathway, or both. As previously demonstrated, the DEESLL signal resides at residues 19–24, and variants of Chs3p carrying mutations in this signal engage in the alternative exocyitc pathway. Thus, if the signal required for exomoer-dependent traffic resided within this region any trafficking defect imparted by deleting it may have been masked by the transit of Chs3p through the alternative exocytic pathway. To test this we expressed Δ15Chs3 and Δ25Chs3 in *chs3Δ chs6Δ* cells and assayed the calcofluor phenotype. Cells expressing either wt Chs3p or Δ15Chs3 in the *chs3Δ chs6Δ* strain were resistant to calcofluor (not shown). This demonstrated that the first 15 amino acids of Chs3p contained no information required for intracellular retention of the protein and thus Δ15Chs3 arrives at the PM solely by the exomer-dependent pathway. In contrast to this, the absence of the DEESLL signal in Δ25Chs3 allowed this protein to access the PM via the alternative exocytic pathway, as confirmed by the calcofluor-sensitive phenotype (not shown). This complicated our ability to test the Δ25Chs3 N-terminal truncation mutant for dependence on exomer. To obviate this problem and determine if Δ25Chs3 relied on both the exomer and alterantive exocytic pathways or only one, the W391R mutation, which blocks access to the alternate route, was introduced into the truncation constructs. In *chs3Δ* cells, expression of Δ15Chs3-W391R conferred a calcofluor-sensitive phenotype, confirming that access of this variant to the PM was independent of the alternative exocytic pathway, and thus reliant on the exomer pathway (Figure 5A) In contrast, cells expressing Δ25Chs3-W391R in a *chs3Δ* strain were resistant to calcofluor, demonstrating that Chs3p lacking the first 25 residues was transported to the PM strictly through the alternative pathway and, due to the loss of residues 16–25, had lost its capacity to traffic via the exomer route (Figure 5A). This result indicated that residues between 16 and 25 of Chs3p were those required for exomer-dependent transport.

To further define the exomer signal within this region, we mutated individual residues to alanine in the context of Chs3-W391R and tested the phenotype of these mutants in a *chs3Δ AP-1Δ* background. The choice of this strain was motivated by our desire to ensure that the alternative pathway would be fully accessed by the different mutants. The expression of Chs3-W391R in *chs3Δ AP-1Δ* cells resulted in calcofluor-sensitivity because Chs3p employed the exomer pathway (Figure 5B, top row). However, when a *chs6Δ* mutation was introduced, cells became calcofluor-resistant because neither the exomer pathway nor the alternative pathway was accessed (Figure 5B, second row). This was the phenotype that we wished to phenocopy by specifically mutating the exomer sorting signal in Chs3-W391R. Of the individual point mutants, only Chs3-D19AW391R and Chs3-E21AW391R showed calcofluor-resistance when expressed in the *chs3Δ AP-1Δ* strain (Figure 5B), suggesting that these residues comprised the exomer sorting signal.

We confirmed that the calcofluor phenotype shown by the Δ15Chs3-W391R, Δ25Chs3-W391R and Chs3-D19AE21A-W391R mutants correlated with a defect of transport to the PM by membrane fractionation on sucrose/EDTA gradients. Whereas the Δ15Chs3-W391R protein co-fractionated with the PM marker Pma1p, Δ25Chs3-W391R and Chs3-D19AE21AW391R failed to populate these fractions (Figure 5C). Finally, we observed that targeted deletions of amino acid residues 50–60 and 60–170, comprising the remainder of the N-terminal cytosolic domain of Chs3p, failed to reveal additional information necessary for exomer-dependent traffic. Deletion of residues 41–50 destabilized Chs3p, preventing us from testing this region (not shown).

### D19 and E21 Define a DXE Motif Necessary for the Interaction of Chs3p with Chs5p

**Figure 6:**
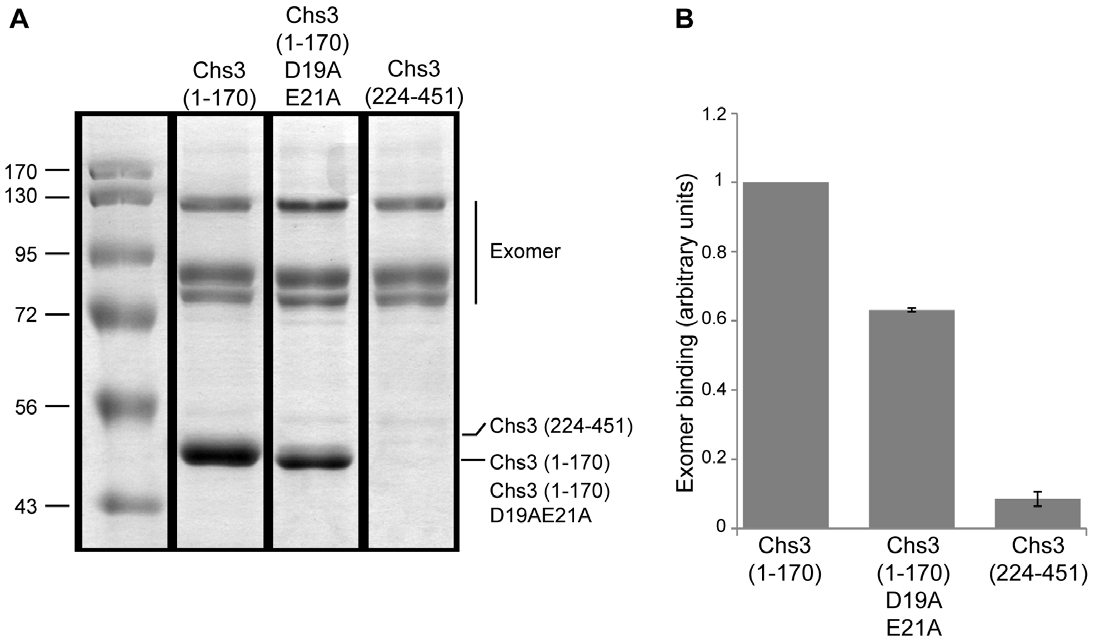
Mutation of D19 and E21 impairs the *in vitro* binding of Chs3p with exomer. (A) Constructs corresponding to the full N-terminal cytosolic tail of Chs3p (Chs3 (1–170)), with and without the double substitution D19AE21A, or to the second cytosolic part of Chs3p (Chs3 (224–451)) were expressed in *E. coli* and purified as soluble GST-fusions. His-Chs5 and His-exomer complexes (expressed and purified from baculovirus infected culture cells [31]) were immobilized on Ni-NTA beads and incubated with 150 ug/ml purified GST-Chs3 fragments at RT for 30 min. Beads were washed and bound proteins were eluted with sample buffer followed by SDS-PAGE and Coomassie Blue staining. All samples shown were analyzed in the same gel. The images of each pertinent lane were cropped and reassembled. (B) The amount of each co-bound Chs3p fragment was quantified and normalized against the amount of Chs5p present in the same lane. The ratios of the amount of co-bound D19AE21A and 224–451 were normalized against the ratio of the wt pull-down, which was arbitrarily set to 1. The values from two independent experiments were averaged and graphed. Error bars represent standard deviations.

We hypothesized that residues D19 and E21 were required for the transport of Chs3p through the exomer pathway and thus would promote the recognition of Chs3p by the exomer complex [30], [31]. To test this, we initially attempted experiments utilizing the same strategy we employed to confirm a role of the DEESLL signal in interaction between Chs3p and the AP-1 complex: in vivo crosslinking followed by purification of TAP-tagged Chs5p. Using this method, we previously showed that Chs3p co-purifies with Chs5-TAP [30], however, experiments testing for reduced co-purification of a Chs3-D19AE21A mutant with Chs5-TAP yielded inconsistent results. We therefore, employed an alternative strategy in which we examined the binding of different regions of Chs3p, purified as soluble GST fusions, to purified His-tagged Chs5p or the entire His-tagged exomer complex immobilized on Ni-NTA beads. Whereas the construct corresponding to the second cytosolic domain of Chs3p (Chs3 (224–451)) bound poorly to the exomer complex, Chs3p (1–170), corresponding to the full N-terminal cytoplasmic tail of Chs3p, showed a robust interaction. Mutation of the key di-acidic motif in the N-terminus of Chs3p (1–170D19AE21A) reproducibly reduced the binding of this domain to the immobilized exomer components. In the case of experiments using only His-tagged Chs5p as bait the reduction ranged from 42–67% of the level of binding of the wt fragment (not shown). Accordingly, in the case of similar experiments in which the complete exomer complex, including His-Chs5, was used the level of Chs3-D19AE21A binding was reduced to 62–63% of wt. (Figure 6A and 6B).

## Discussion

The current model for trafficking of Chs3p within the late secretory pathway proposes a key sorting decision at the TGN: transport to the PM through the exomer pathway or transport to and from the EE to maintain an intracellular reservoir. Mutations in AP-1, AP-3, GGA1/2, Ent3/5 or the DEESLL signal cause Chs3p to be rerouted to the PM via a poorly understood alternative pathway, independent of exomer. Here we showed that distinct events in the post-Golgi traffic of Chs3p are controlled by multiple sorting determinants.

In our studies of the intracellular transport of Chs3p, we employed a genetic selection for an intracellular sorting signal and identified four alleles of *CHS3* that define the DEESLL signal in the N-terminus of Chs3p. This signal matches the consensus sequence for AP-dependent signals in mammalian and yeast cells [6]. Importantly, mutation of this signal exerts the same effect as mutation of the AP-1 complex: Chs3p is able to access the alternative exocytic pathway and reach the PM in *chs6Δ* cells. Additionally, biochemical experiments confirmed that AP-1 physically interacts with Chs3p via the DEESLL signal (Figure 2). Thus the DEESLL signal constitutes the first [D/E]XXXL[L/I/V] signal found to be dependent on AP-1 in yeast cells. Furthermore, the DEESLL signal is remarkable in that it also harbors a DXE motif used by the exomer complex for delivery of Chs3p to the PM. Whereas residues D19 and E21 are most important for the exomer-dependent exocytic pathway, residues L23 and L24 are most critical for the AP-1-dependent intracellular pathway.

The involvement of AP-3 in trafficking Chs3p was surprising given our previous conclusion to the contrary [33]. The difference may be due to strain background differences, which may alter the sensitivity of cells to calcofluor and ½ YPD media. We previously used a strain from the SEY6210 background to test the involvement of AP-3 in Chs3p trafficking [33], but have used strains from the YPH499 background in this study. The involvement of AP-3 in Chs3p trafficking was also surprising because AP-3 is implicated in delivering the proteins ALP and Vam3p from the TGN directly to the vacuole, bypassing the endosomal system [45], [46], whereas Chs3p does not obviously localize to the vacuole. We were unable to trap Chs3p in the vacuole in a *vac7Δ* mutant, which is impaired in a vacuolar retrieval pathway, even when *CHS6* was also deleted to restrict Chs3p to intracellular compartments (not shown). Thus our findings raise the possibility that AP-3 may transport Chs3p between the TGN and endosomes instead of to the vacuole. The mechanism by which AP-3 transports Chs3p is unknown, but AP-3 also acts on [D/E]XXX[L/V/I] signals [6], and thus may also have some interaction with the DEESLL signal. This possibility remains to be tested. Finally it is important to note that AP-3 is also required for the intracellular retention of the other known exomer-dependent PM protein, Fus1p (personal communication, Robyn Barfield).

In our studies of the alternative exocytic pathway we showed by two independent methods (calcofluor phenotype and sucrose gradient fractionation) that mutations in residues R374 and W391 block access of Chs3p to the route responsible for targeting Chs3p to the PM independently of the exomer complex. The exact route of this pathway remains to be determined. Depending on the directionality of the trafficking event mediated by AP-1, the W391 signal may control transport of Chs3p from the TGN to the EE, from the EE to the PM, or from the TGN to the PM. Determining where the APs function in Chs3p transport has been difficult, thus we have not yet been able to distinguish these possibilities.

Chs5p and its partners present many features consistent with a function as coat proteins for the transport of transmembrane proteins from the TGN to the PM: (1) In *chs5*Δ mutants, Chs3p and Fus1p are not transported to the PM and are retained within the cell [47], [48], (2) Chs5p, Chs6p and three Chs6p paralogs assemble into the ∼1 MDa exomer complex that binds Chs3p *in vivo* and forms a spiky coat on synthetic liposomes *in vitro* [30], [31] and (3) Chs5p and the exomer components Bch1p and Bud7p are required for the exocytic trafficking of Fus1p to the PM [28]. We show here that the interaction of exomer with Chs3 *in vitro* is dependent on the residues D19 and E21. Furthermore, these residues are essential for the transport of Chs3p from the TGN to the PM. Together these results suggest that the di-acidic motif D19 and E21 of Chs3p define a cytosolic sorting determinant decoded by exomer for transport from the TGN to PM. It is worth noting that the D19A E21A mutation, although conferring strong calcofluor resistance in our genetic analysis of the exomer motif (Figure 5B), impairs only by ∼40% the direct binding to exomer when tested with recombinant proteins (Figure 6B), illustrating that the effect of this mutation on the binding to exomer is more stringent in vivo in the context of the full-length chs3p than in vitro.

It is also worth noting that DXE motifs can also serve as ER-export signals [49], and therefore the possibility of residues 19DEE21 acting in ER exit of Chs3p must be considered. Analysis of Chs3-GFP lacking the DESSLL signal, and thus lacking residues 19DEE21, does not reveal obvious entrapment of Chs3-GFP in the ER, indicating that the elimination of the DXE signal does not confer a major ER export defect (not shown). We have not made measurements of the kinetics of Chs3p exit from the ER, and therefore are unable to formally rule out the possibility that the D19AE21A mutation causes a slight ER exit defect. However, based on the lack of obvious ER accumulation in combination with the strong trafficking defects revealed by our calcofluor and fractionation studies, we believe the bulk of the defect is in trafficking from the Golgi to the PM. Finally, the observation of the proximity within the N-terminal domain of Chs3p of the motifs for the exomer pathway and the AP-1 recycling pathway raises the interesting possibility of regulation of Chs3p traffic through competitive interactions between these two signals and their respective coat proteins. Such regulation would be consistent with the known trafficking events that mediate specific subcellular delivery of Chs3p within the late secretory pathway. Chitin ring formation early in the cell cycle is controlled by the timed transport of Chs3p from the TGN to the PM by the exomer complex [50]. Moreover, cell stress, such as high temperature, causes similar relocation of the internal pool of Chs3p to the PM [40]. This environmental regulation is dependent on the action of protein kinase C, Pkc1p, which, among many other functions, is necessary for the phosphorylation of Chs3p [40]. In mammalian cells, the phosphorylation of a serine or threonine is known to influence the traffic of membrane proteins through the TGN/endosomal system by interfering with the activity of a nearby sorting signal [3], [51]. At least three serines (S22, S26, and S29) are located in the vicinity of the DESSLL signal, and the phosphorylation state of these residues may influence the affinity of this region for different coat proteins. However, preliminary characterization of the DEESLL signal suggests that modification of these residues is not a positive determinant to engage Chs3p in either the exomer pathway or the AP-1 pathway, as the single substitutions S22A, S26A and S29A did not block access to either of these transport pathways (Figure 5B, 5C). Nevertheless, the additive contribution of these mutations, or a negative influence caused by the phosphorylation of these residues, or the contribution of more distal serine or threonine residues cannot be excluded. Further studies of the traffic of Chs3p should continue to present opportunities to investigate the regulation of vesicular transport in the late secretory pathway of eukaryotic cells.
